# First record of the genus *Olepa* Watson, 1980 from China (Lepidoptera, Erebidae, Arctiinae, Arctiini)

**DOI:** 10.3897/BDJ.10.e78167

**Published:** 2022-02-14

**Authors:** Yulong Zhang, Siyao Huang, Min Wang

**Affiliations:** 1 South China Agricultural University, Guangzhou, China South China Agricultural University Guangzhou China

**Keywords:** Guangdong, Hainan, molecular analysis, venation, genitalia, synonym

## Abstract

**Background:**

The tribe Arctiini is a species-rich tribe of the subfamily Arctiinae of the family Erebidae. The genus *Olepa* Watson, 1980 is distributed in the Oriental and Palearctic Regions and the diversity reaches its peak in south Asia.

**New information:**

We herein describe the first record of the genus *Olepa* from China and re-describe *Oleparicini* (Fabricius, 1775), together with illustrations of its adult and male genitalia. Furthermore, based on an analysis of 658-bp COI barcoding sequences, together with morphological studies, we consider that *Olepaschleini* Witt et al., 2005 **syn. n.** is a new synonym of *O.ricini*.

## Introduction

The genus *Olepa* was originally established by Watson (1980) as a replacement name for the genus *Alope* Walker, 1855 [preoccupied by *Alope* White, 1847 (Crustacea)] with *Alopeocellifera* Walker, 1855 as the type species. Orhant (1986) carried out a taxonomic review of the *Oleparicini* Fabricius, 1775 species group, restored the specific status of *O.ocellifera* Walker and *O.clavatus* Swinhoe and described four new species from southern India and Sri Lanka. Subsequently, Orhant (2000) added a new species from north-eastern India and divided the genus *Olepa* into two species groups, based on differences in male genitalia. Subsequent studies revealed three additional species from western Asia, India and Indochina (Witt et al. 2005, Dubatolov 2011, Orhant 2012) and, later, Singh and Singh (2013) revised the internal structure of the genus and described four subgenera (*Ricinia*, *Pseudoolepa*, *Orhanta* and *Cornutia*). Two species, namely *O.neumuthi* and *O.toulgoeti*, were treated as the synonyms of *O.ricini* and *O.clavatus*, respectively. Recently, Kalawate et al. (Kalawate et al. 2020a, Kalawate et al. 2020b) described further five new taxa from India and restored *O.toulgoeti* as a valid species. Therefore, currently the genus *Olepa* is comprised of four subgenera, 14 species and two subspecies, which are widely distributed from western Asia to Indochina, while without any record from China. However, during a study of the Lepidoptera collection in the South China Agricultural University, we discovered four specimens of this genus that represent the first records of *Olepa* from China and they belong to the same species, *O.ricini*. Moreover, based on our molecular and morphology studies, we concluded that *O.schleini*, which was described from Israel, should be a synonym of *O.ricini*. Thus, the current paper is dedicated to the report of the first record of the genus Olepa from China with synonymising of *O.schleini* with *O.ricini*.

## Materials and methods

Morphological study. We photographed the adults using a Sony DSC-RX100 v1.00 camera. To study the wing venation, the wings were removed from the thorax and cleaned with a 1:1 mixture of bleaching liquid. A Nikon D750 camera was used to photograph the venation. Abdomens were removed and macerated in hot 10% sodium hydroxide (NaOH) solution for examination of male genitalia, photographs of which were taken under a Keyence VHX-5000 digital microscope. Terminology of adult and genitalia follows Fang (2000).

Molecular phylogenetic analysis. We selected 19 samples representing the species of *Olepa* as the ingroup and, for the outgroup, we used *Trischalisaureoplagiata* and *Cyclosiellaspiralis* (two species of the tribe Lithosiini of the subfamily Arctiinae). We sequenced four specimens from China and the remaining sequences were obtained from GenBank and Bold Systems. DNA was extracted from two or three legs of dried adult specimens using a TIANamp Genomic DNA Kit (Tiangen, Guangzhou, China) following the manufacturer’s instructions. We amplified a single mitochondrial gene (a 658-bp fragment of COI) using the general primers 1490-2198 (Folmer et al. 1994). The amplification protocol follows Hou et al. (2021). Sequences were aligned using Clustal W (Thompson 1997) and edited manually using MEGA 7.0 (Kumar et al. 2016). Maximum Likelihood analyses, shown in Fig. 4, were performed using IQ-tree v. 1.6.12 (Trifinopoulos et al. 2016) with the branch support values evaluated by 1000 ultrafast bootstrap (UFBS) replicates (Trifinopoulos et al. 2016) on the web server (http://iqtree.cibiv.univie.ac.at/), the best-fit model used being automatically selected by IQ-TREE (TIM2+F+I chosen according to Bayesian Information Criterion). New sequences have been deposited in GenBank (Table 1).

## Taxon treatments

### Olepa ricini

(Fabricius, 1775)

7F131776-AEE6-57B1-A7C1-524B2EEEE8C9

Bombyx ricini Fabricius 1775: 583 (Type locality: India)
Alope ricini Moore, 1882: 70
Pericallia ricini Hampson, 1901, 350
Olepa ricini Watson, 1980:133
Olepa (Ricinia) ricini ; Singh & Singh, 2013:276
Olepa neumuthi Orhant, 2012:61, synonymised by Singh & Singh, 2013
Olepa schleini Witt et al., 2005:102 (Type locality: Israel, Tel Aviv North), **syn. nov.**

#### Materials

- **Type status:** Other material. **Occurrence:** recordedBy: S.Y. Huang; sex: 1 male; lifeStage: adult; occurrenceID: SCAU:Ole02; **Taxon:** scientificName: *Oleparicini* (Fabricius, 1775); order: Lepidoptera; family: Erebidae; genus: Olepa; taxonRank: species; taxonomicStatus: accepted; **Location:** country: China; stateProvince: Guangdong; county: Guangzhou; locality: campus of South China Agricultural University; **Event:** eventDate: 14-Sep-2021
- **Type status:** Other material. **Occurrence:** recordedBy: S.Y. Huang; sex: 1 male; lifeStage: adult; occurrenceID: SCAU:Ole01; **Taxon:** scientificName: *Oleparicini* (Fabricius, 1775); order: Lepidoptera; family: Erebidae; genus: Olepa; taxonRank: species; taxonomicStatus: accepted; **Location:** country: China; stateProvince: Guangdong; county: Guangzhou; locality: campus of South China Agricultural University; **Event:** eventDate: 24-Sep-2020
- **Type status:** Other material. **Occurrence:** recordedBy: Y.X Hou; sex: 1 males; lifeStage: adult; occurrenceID: SCAU:Ole03; **Taxon:** scientificName: *Oleparicini* (Fabricius, 1775); order: Lepidoptera; family: Erebidae; genus: Olepa; taxonRank: species; taxonomicStatus: accepted; **Location:** country: China; stateProvince: Guangdong; county: Guangzhou; locality: campus of South China Agricultural University; **Event:** eventDate: 1-Oct-2021
- **Type status:** Other material. **Occurrence:** recordedBy: L.P. Zhou; sex: 1 males; lifeStage: adult; occurrenceID: SCAU:Ole04; **Taxon:** scientificName: *Oleparicini* (Fabricius, 1775); order: Lepidoptera; family: Erebidae; genus: Olepa; taxonRank: species; taxonomicStatus: accepted; **Location:** country: China; stateProvince: Hainan; locality: Yinggeling Natural Reserve; **Event:** eventDate: 13-Jul-2020
- **Type status:** Other material. **Occurrence:** recordedBy: P. Qin; sex: 1 male; lifeStage: adult; occurrenceID: SCAU:Ole05; **Taxon:** scientificName: *Oleparicini* (Fabricius, 1775); order: Lepidoptera; family: Erebidae; genus: Olepa; taxonRank: species; taxonomicStatus: accepted; **Location:** country: China; stateProvince: Guangdong; county: Guangzhou; locality: campus of South China Agricultural University; **Event:** eventDate: 14-Oct-2021

#### Description

**Figure 1.**
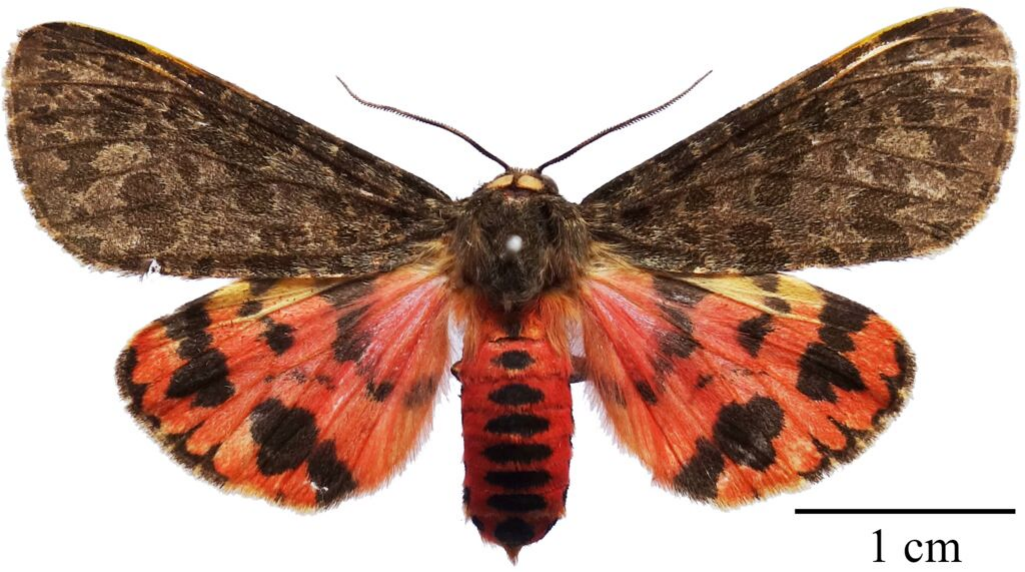
Adult of *Oleparicini* (Fabricius, 1775) (♂).

**Figure 2.**
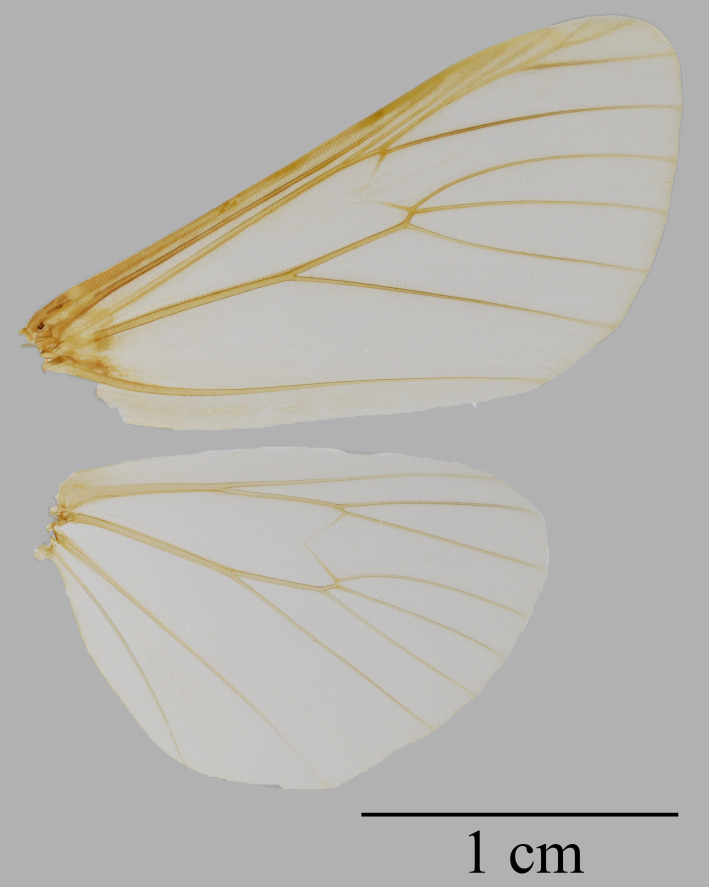
Wing venation of *Oleparicini*.

Male (Fig. 1): Length of forewing 20 mm. Antenna, head and thorax brownish-grey; tegula yellow; patagium covered with brownish-grey hair; abdomen scarlet with elongated black spots of various length on the dorsal side. Forewing ground colour dark brown, with six transverse bands comprised of irregular blackish-brown spots. Cilia chequered. Hind-wing ground colour red with black patterns; antemedian band extending from costal zone to dorsum, gradually narrowing; median band obsolete, extending from costa to upper angle of cell; postmedian band thick, running from costa to tornus and interrupted in cell M_2;_ marginal line serrate, extending from apex to vein CuA_1_. Venation (Fig. 2): Forewing: Sc free, extending to 2/3 of costa, R_1_ extending from near the upper corner of the median cell, R_2_, R_3_, R_4_ and R_5_ stalked, R_3_ and R_4_ stalked; M_1_ extending from the upper corner of the median cell, M_2_, M_3_ and CuA_1_ originating from the lower corner of the median cell; CuA_2_ originating from almost the mid-point of the cubitus; 1A originating from wing base. Hind-wing: Sc+R_1_ originating from nearly the mid-point of the upper edge of the median cell; Rs arising beyond the upper angle of the cell, M_1_ arising just at the upper angle of the cell; M_2_, M_3_ and CuA_1_ all arising near or at the lower angle of the cell; CuA_2_ arising from nearly the midpoint of cubitus; 2A and 1A free.

**Figure 3.**
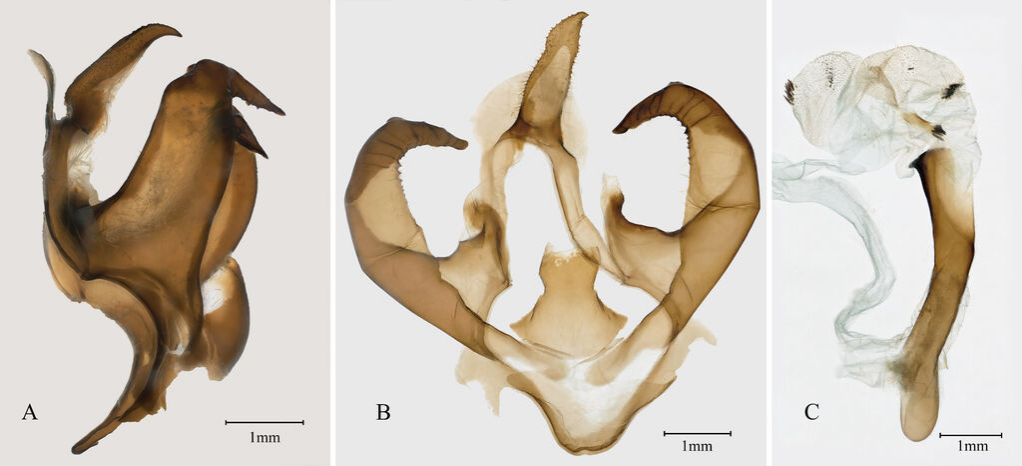
Male genitalia of *Oleparicini*. **A.** lateral view of genitalia capsule; **B.** genitalia capsule flattened; **C.** phallus.

Male genitalia (Fig. 3): uncus relatively long and broad and gradually narrowed towards the distal end; tegumen narrow with a semi-oval dorsal plate; juxta shield-like; saccus nearly U-shaped and short; valva moderately broad with its tip curved inwardly; phallus long and slightly S-like curved, carinal plate with a horn-shaped protrusion, vesica broad, with several clusters of small cornuti.

Female: unknown.

#### Distribution

China (new record), Thailand, India, Israel.

## Discussion

**Figure 4.**
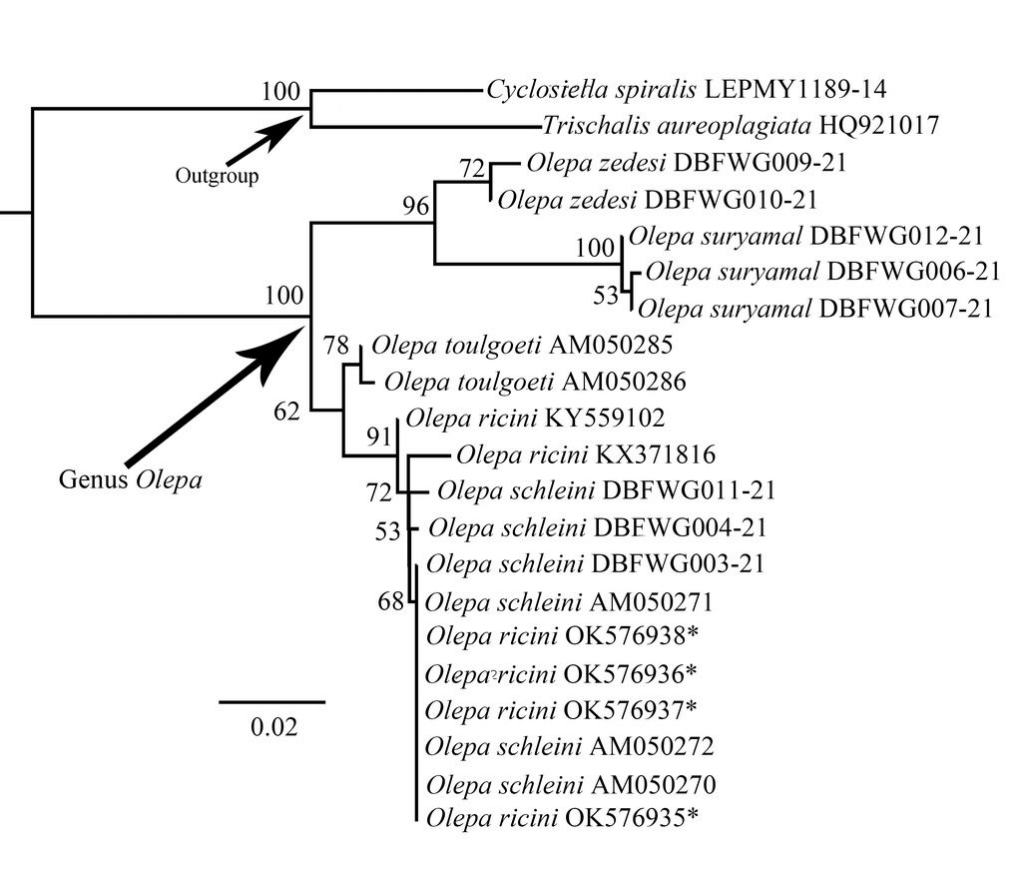
Maximum Likelihood (ML) tree for the species of *Olepa*, based on the COI gene. *OK576935 - OK576938 were collected in China.

The Chinese population of *Olepa* is morphologically similar to *O.schleini* and *O.ricini*. These two species were supposed to be distinguished from each other mainly by the differences in the tip of the valva, i.e. tip rounded in the former and more acute in the latter, together with a great genetic difference in COI sequences (Witt et al. 2005). In addition, another difference was also mentioned in the original description, that is the dark spots on forewing upper side are not surrounded by pale rings in the former, while the pale rings are usually present in the latter. However, based on our examined specimens, we found that the tip of the valva can be quite variable when viewed from different aspects and even flattened, hence we concluded this character cannot be regarded as a stable difference. Moreover, after conducting the BLAST procedure of the COI sequences of *O.ricini* (GenBank accession numbers AM050280-AM050284) used by Witt et al. (2005) in GenBank, we found that these sequences are actually closer to those of certain species in the family Geometridae rather than other members of Arctiinae of the family Erebidae. It is very likely that these COI sequences of *O.ricini* are wrong and are not the true ones of this species. This result can also explain why the genetic difference between *O.ricini* and *O.toulgoeti* was also as large as 18.2% to 18.5%, but the difference between *O.schleini* and *O.toulgoeti* was only 1.6% to 1.8%, despite the fact that *O.schleini* and *O.ricini* are morphologically closer. Based on our analysis, the genetic difference between Chinese *Olepa* sp. and Israeli *O.schleini* is 0%, the difference between two true Indian *O.ricini* (GenBank accession numbers KX371816 and KY559102) and Israeli *O.schleini* varies from 0.3% to 0.9% and the difference between Chinese *Olepa* sp. and Indian *O.ricini* also varies from 0.3% to 0.9%. Considering the fact that the genetic difference between the morphologically dissimilar *O.ricini* and *O.toulgoeti* is only 1.6% to 1.8% and the difference found between Indian *O.ricini* and Israeli *O.schleini* is smaller than 1%, we believe that all the three populations of *O.ricini* and *O.schleini* mentioned above should be regarded as conspecific. As for the difference in wing pattern, according to Orhant (1986), the lectotype of *O.ricini* housed in the Natural History Museum of Denmark is a female without abdomen (plate 1, figs. 4 and 5 in Orhant 1986) and the holotype of *O.schleini* is a male deposited in Museum Witt Munich, but the adult of this specimen was not specified in the plates in Witt et al. (2005). By comparing the female lectotype of *O.ricini* and illustrations of males of this species from various publications with males and females of *O.schleini* figured in Witt et al. (2005), we found no significant difference between these individuals and, interestingly, the pale rings are mostly reduced in the lectotype of *O.ricini*, similar to the situation in *O.schleini*. Hence, we concluded that this character is most probably due to individual variation. Thus, we herein present the following synonym: *Olepaschleini* Witt et al., 2005 **syn. n.** = *Oleparicini* (Fabricius, 1775) and the Chinese *Olepa* sp. should be also identified as *O.ricini*.

## Supplementary Material

XML Treatment for Olepa ricini

## Figures and Tables

**Table 1.** Voucher information and accession numbers for the specimens in this study.

Taxon	Locality	Date	Voucher Number	Accession Number
*Oleparicini*	China	24-Ⅸ-2020	SCAU Ole01	OK576935
*Oleparicini*	China	14-Ⅸ-2021	SCAU Ole02	OK576936
*Oleparicini*	China	1-Ⅹ-2021	SCAU Ole03	OK576937
*Oleparicini*	China	13-Ⅶ-2020	SCAU Ole04	OK576938
*Olepaschleini*	Israel	19-Ⅶ-2005	DNATAX02723	AM050270*
*Olepaschleini*	Israel	19-Ⅶ-2005	DNATAX02724	AM050271*
*Olepaschleini*	Israel	19-Ⅶ-2005	DNATAX02725	AM050272*
*Olepaschleini*	India	07-Ⅹ-2017	ZSI_WRC_L_2029	DBFWG011-21^+^
*Olepaschleini*	India	24-Ⅱ-2019	MOGSJ 03	DBEM003-20^+^
*Olepaschleini*	India	04-Ⅶ-2019	ZSI_WRC_L_2028	DBFWG004-20^+^
*Olepasuryamal*	India	07-Ⅹ-2017	ZSI_WRC_L_2148	DBFWG006-21^+^
*Olepasuryamal*	India	07-Ⅹ-2017	ZSI_WRC_L_2149	DBFWG007-21^+^
*Olepasuryamal*	India	07-Ⅹ-2017	ZSI_WRC_L_2150	DBFWG012-21^+^
*Olepazedesi*	India	23-Ⅷ-2017	ZSI_WRC_L_2154	DBFWG009-21^+^
*Olepazedesi*	India	17-Ⅷ-2017	ZSI_WRC_L_2155	DBFWG010-21^+^
*Oleparicini*	India	N/A	N/A	KY559102*
*Oleparicini*	India	05-ⅩⅡ-2012	RO_Olepric-1	KX371816*
*Olepatoulgoeti*	India	4-Ⅳ-1997	DNATAX02741	AM050285*
*Olepatoulgoeti*	India	4-Ⅳ-1997	DNATAX02742	AM050286*
*Trischalisaureoplagiata*	Australia	15-Ⅲ-1992	10ANIC-00599	HQ921017*
*Cyclosiellaspiralis*	Malaysia	18-Ⅴ-2014	BIOUG14394-C09	LEPMY1189^+^

* Indicates that the gene sequence was downloaded from NCBI, ^+^ Indicates that the gene sequence was downloaded from Bold Systems.
